# Molecular gated nanoporous anodic alumina for the detection of cocaine

**DOI:** 10.1038/srep38649

**Published:** 2016-12-07

**Authors:** Àngela Ribes, Elisabet Xifré -Pérez, Elena Aznar, Félix Sancenón, Teresa Pardo, Lluís F. Marsal, Ramόn Martínez-Máñez

**Affiliations:** 1 Instituto Interuniversitario de Investigaciόn de Reconocimiento Molecular y Desarrollo Tecnolόgico (IDM). Universitat Politècnica de València, Universitat de València, Departamento de Química, Universitat Politècnica de València, Camino de Vera s/n, 46022, Valencia, Spain; 2 CIBER de Bioingeniería, Biomateriales y Nanomedicína (CIBER-BBN); 3 Departamento de Ingeniería Electrónica, Eléctrica y Automática, Universidad Rovira i Virgili, Avda. Països Catalans 26, 43007, Tarragona, Spain

## Abstract

We present herein the use of nanoporous anodic alumina (NAA) as a suitable support to implement “molecular gates” for sensing applications. In our design, a NAA support is loaded with a fluorescent reporter (rhodamine B) and functionalized with a short single-stranded DNA. Then pores are blocked by the subsequent hybridisation of a specific cocaine aptamer. The response of the gated material was studied in aqueous solution. In a typical experiment, the support was immersed in hybridisation buffer solution in the absence or presence of cocaine. At certain times, the release of rhodamine B from pore voids was measured by fluorescence spectroscopy. The capped NAA support showed poor cargo delivery, but presence of cocaine in the solution selectively induced rhodamine B release. By this simple procedure a limit of detection as low as 5 × 10^−7^ M was calculated for cocaine. The gated NAA was successfully applied to detect cocaine in saliva samples and the possible re-use of the nanostructures was assessed. Based on these results, we believe that NAA could be a suitable support to prepare optical gated probes with a synergic combination of the favourable features of selected gated sensing systems and NAA.

The combination of different inorganic solids with organic compounds has resulted in the preparation of an almost unlimited number of new supports with a wide range of new functionalities. In this field, interesting materials are gated nano-devices that can deliver an entrapped cargo in the presence of certain external stimuli^1^,^2^,^3^,^4^. In most cases gated materials are based on a suitable inorganic support and a switchable ensemble capable of being “opened” or “closed” on command. In fact gated nanochemistry is a highly topical and rapidly developing tool that is demonstrating the possibility of achieving new advanced pre-designed functions by means of mass transport control. In fact in the last few years, nano-containers that bear gated scaffoldings have proved excellent candidates for the design of controlled-release “nanomachines” at different levels. Gated materials have been used mainly in drug delivery, although some examples have also been reported for sensing applications^5^,^6^,^7^,^8^. For the latter, the approach involves loading the porous support with a reporter and using a capping mechanism in such a way that cargo delivery is triggered by the presence of the target analyte. According to this principle, gated systems able to respond to anions^9^,^10^, cations^11^,^12^,^13^, neutral molecules^14^,^15^,^16^,^17^ or biomolecules^18^,^19^,^20^ have been recently developed. Most of these sensing materials are based on the use of mesoporous silica nanoparticles thanks to their well-known properties, such as large load capacity, biocompatibility and functionalization ease. However, the use of nanoparticulated supports for sensing has some drawbacks. In particular, nanoparticles are not easy to handle, can be harmful if breathed in or deposited on skin, and can sometimes not form uniform suspensions to obtain highly reproducible sensing systems. Despite these problems, the implementation of gated ensembles for sensing applications on other nanostructured supports has not yet been fully developed.

From a different point of view, nanoporous anodic alumina (NAA) has emerged as a competitive support in nanotechnology for a significant number of applications^21^,^22^,^23^. Moreover, preparation of NAA is easy, cost-effective and easily up-scalable by well-known production techniques^24^. Nanotechnological applications of this material can be found in a wide variety of fields, such as energy, nanofabrication or biotechnology^25^,^26^,^27^,^28^. One interesting advantage of NAA over other systems for cargo release is that their structure is stable and does not degrade or erode in aqueous solutions, which may contribute to the development of robust reproducible devices, most of them for biosensing applications^29^,^30^,^31^. NAA is also biocompatible, and can be calcined and reused several times, which prolongs the support’s lifespan.

In this context we were interested in studying the possible use of NAA as support to design gated materials for sensing applications. To develop our idea, we selected an aptamer-based gating mechanism. Aptamers have been widely recognised as versatile signal-transducing elements for sensing platforms^32^,^33^,^34^. Aptamers are DNA sequences with high selectivity and affinity for certain target proteins, small molecules or ions^35^,^36^,^37^. Their production is based on an *in vitro* selection method called SELEX (systematic evolution of ligands by exponential enrichment)^38^,^39^,^40^ and their use has revolutionised the sensing field as ultrasensitive systems have been obtained to detect a number of different analytes^41^,^42^. In the particular field of gated materials, Özalp and co-workers described the first aptamer-based gating mechanism capable of selectively releasing fluorescein in the presence of ATP^43^. Based on this first example, some other reports based on aptamers and gated materials have been reported for ATP^44^, potassium^45^, adenosine^46^ and thrombin^47^, which indicate the high potential of aptamers as capping systems to prepare highly selective and sensitive probes.

We herein selected cocaine as the target analyte, which is a powerful addictive stimulant drug isolated from coca plant leaves. Its consumption causes a short-lived intense high, which is immediately followed by the opposite, e.g., intense depression, edginess and dependence. It is one of the commonest illegal drugs consumed worldwide and the design of easy-to-use detection systems for cocaine is very important. For this reason, many researchers have been involved in developing new methods to detect cocaine^48^. Immunoassay techniques are the first option for the rapid screening of illicit drugs^49^. However, these methodologies are sometimes not very selective and require positive confirmation by other procedures. Liquid or gas chromatography coupled with a mass spectrometry detector is the most widely used procedure to identify cocaine^50^. Although the limit of detection (LOD) of this method is very low, this technique is not widely accessible, is not portable, is time-consuming and requires trained personnel. Hence aptamers have also been used to develop cocaine-sensing systems. For instance, Soh *et al*.^51^ developed a method based on the use of target-specific DNA aptamers which generates an electrochemical signal in response to cocaine coupled with a microfluidic detection system. Cai *et al*.^52^ fabricated a sandwich biosensor by using fragments of the cocaine aptamer immobilised in a gold electrode and on the surface of tris (2,2′-bipyridyl)ruthenium(II)-doped silica nanoparticles, which gave an electroluminescent signal if cocaine was present. Liu *et al*. designed a simple colorimetric method^53^ with an aptamer attached to the surface of gold nanoparticles, which aggregated when cocaine was present. Finally, Yu *et al*.^54^ designed a label-free method in which cocaine selectively induced the polymerisation of an oligonucleotide that detached from a graphene oxide (GO) platform and brought about a change in the support’s fluorescence.

Given the background of these fields (i.e. gated materials, optical sensors design, aptamers and NAA supports), we became interested in combining the use of NAA and gating concepts to prepare a new cocaine-sensing system. The design of the probe is depicted in Fig. 1. The NAA support was loaded with a fluorescent reporter (rhodamine B) and functionalized with a short single-stranded DNA. Then pores were capped by the subsequent hybridisation of a specific cocaine aptamer. The capping DNA sequence was expected to be bulky enough to block pores and to inhibit dye delivery. Presence of the target analyte (i.e. cocaine) in the medium was expected to selectively displace the aptamer, resulting in pore opening and dye release.

## Results and Discussion

### Preparation and characterization of the sensing support

The NAA support was obtained according to reported procedures^55^. Firstly, high purity aluminium sheets were electropolished in a mixture of ethanol and perchloric acid. Then the electropolished aluminium sheets were anodised by a two-step anodisation process with sulphuric acid. During this process, the second anodisation time was crucial to acquire a specific thickness on the aluminium layer (8 μm). To implement the gating mechanism, NAA supports were loaded with rhodamine B dye. Then the external surface was functionalized with (3-isocyanatopropyl)triethoxysilane to yield support **S1**. In another step, the short DNA sequence NH_2_-(CH_2_)_6_-5′-AAA AAA CCC CCC-3′ (**O1**), specifically designed to hybridise with a 3′-TTT TGG GGG G-5′ sequence was covalently attached to **S1** by the formation of urea bonds to give support **S2**. Finally, the single-stranded oligonucleotide 5′-TTT TGG GGG GGG GAG ACA AGG AAA ATC CTT CAA TGA AGT GGG TCT CCA GGG GGG TTTT-3′ (**O2**), which contained the specific sequence of the cocaine aptamer with dissociation constant K_D_ ~20 μM^44^, was used to cap pores by hybridisation with **O1** to yield the final sensing capped support **S3**.

**Figure 2:**
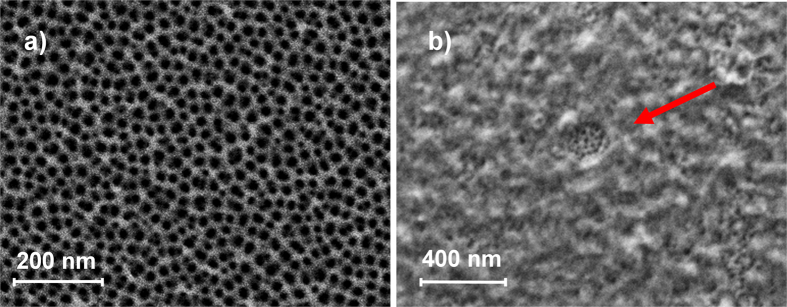
FESEM images of (**a**) parent NAA support and (**b**) support **S3**. Arrow indicates a certain area where the porous framework can be seen below the organic layer.

The starting NAA support was characterised by field emission scanning electron microscopy (FESEM), powder X-ray diffraction and thermogravimetric analyses. All the functionalized supports were also characterised by the same techniques. The nanostructure of the starting NAA support was assessed by FESEM. Representative images showed disordered pores with an average diameter of 8 nm (see Fig. 2a). This disordered structure was confirmed by powder X-ray diffraction (See the Supplementary Information), where only a very strong peak at 2θ = 38°, which corresponded to crystalline aluminium, and a weak and broad peak, which corresponded to amorphous alumina oxide, were found. No more peaks were recorded, which indicated that the support did not present an ordered pore distribution. Supports **S1**, **S2** and **S3** showed a similar powder X-ray diffraction pattern to the initial support. The FESEM images of the **S3** support clearly showed the presence of an organic layer that covered most pores. The presence of this organic layer evidenced the suitable consecutive loading, functionalization and capping steps, while the visualisation of the porous framework in certain areas (see the arrow in Fig. 2b) confirmed the preservation of the nanoporous structure in **S3**.

The functionalization of the NAA support was followed by EDX. As expected, the EDX analysis on the starting NAA gave peaks for aluminium and oxygen, whereas EDX on **S3** also clearly showed the presence of silicon, carbon, nitrogen and phosphorous from the anchored isocyanate moieties and oligonucleotides. The organic content of the different prepared materials was also calculated from the thermogravimetric analysis (see Table 1). An amount of 0.24 mmol/gSiO_2_ of rhodamine B, 0.10 mmol/gSiO_2_ of **O1** and 0.11 mmol/gSiO_2_ of **O2** was determined for **S3**. The high content of the (3-isocyanato)propyl organosilane derivative incorporated into **S1** indicated that functionalization was not only restricted to the aluminol groups on the external surface, which suggested that incorporation of (3-isocyanato)propyl organosilane groups into mesopore entrances and/or external polymerization cannot be ruled out. From these data contents, the molecular ratio of **O1** compared to the (3-isocyanato) propyl moieties in **S3** was 35.4%, while the ratio between **O2** and **O1** was 48.6%, which indicated a good coverage of NAA with the aptamer.

### Sensing support performance

**Figure 3:**
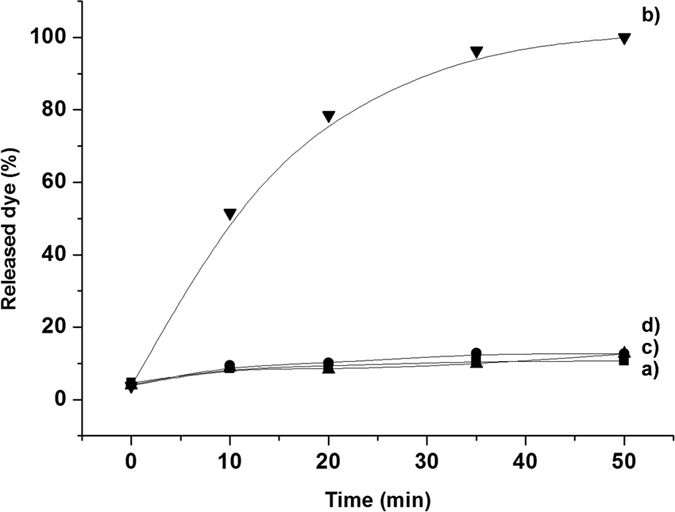
Release of rhodamine B from support **S3** (**a**) in the absence and (**b**) presence of cocaine (1 mM). Release of rhodamine B from support **S4** (**c**) in the absence and (**d**) presence of cocaine (1 mM).

The response of support **S3** to the presence of cocaine was studied in aqueous solution. In a typical experimental, two **S3** supports were immersed in 2.5 mL of hybridization buffer (Tris-HCl 20 mM, MgCl_2_ 37.5 mM, pH 7.5) each. Then 2.5 mL of an aqueous solution of cocaine (1 mM) were added to one of the supports, whereas 2.5 mL of water were added to the second support. Both systems were maintained at 25 °C and aliquots of the supernatant solution were taken at certain times. Cargo release was measured by rhodamine B emission at 585 nm (λ_exc_ = 555 nm). Figure 3 shows the delivery profile of rhodamine B from support **S3** in the presence and absence of cocaine. In the absence of the target analyte (Fig. 3, curve a), poor rhodamine B delivery took place, which is indicative of remarkable pore closure. In contrast when cocaine was present, clear dye delivery to the solution was detected (Fig. 3, curve b). A simple calculation allowed us to determine that the ratio between the cargo released by leakage and that released upon cocaine recognition at a concentration of 0.01 mM after 20 minutes was 8-fold.

In order to assess the specificity of the sequence chosen in our work (**O2**), a NAA support loaded with rhodamine B and capped with a scrambled sequence (**O3**) was prepared (support **S4**) by a similar procedure to that used for **S3**. Our intention on preparing **S4** (that was capped by a DNA sequence that does not present any cocaine affinity) was to demonstrate that the cocaine selective opening of **S3** solid was induced by the aptamer used to cap the pores. In **S3**, selective coordination of cocaine with the aptamer induced selective pore opening and dye release, whereas it was expected that addition of cocaine to **S4** would not result in payload delivery. In fact, as depicted in Fig. 3, the release of rhodamine B from **S4** in the absence (Fig. 3, curve c) and presence of cocaine (Fig. 3, curve d) was the same as that obtained for support **S3** in the absence of the analyte. This result confirmed the requirement of using a specific cocaine-aptamer to cap the NAA support pores to prepare selective probe **S3**.

**Figure 4:**
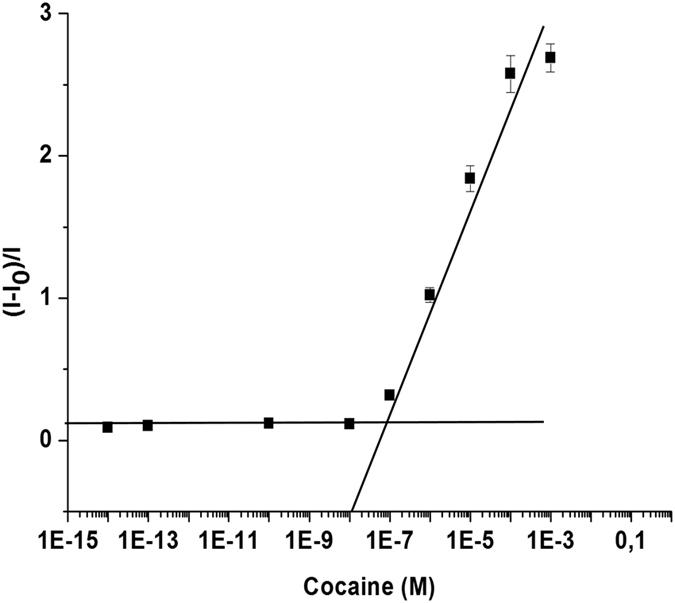
Release of rhodamine B from support **S3** according to the cocaine concentration in Tris-HCl buffer.

In another step, the response of **S3** to different concentrations of cocaine was studied. Following a procedure similar to that described above, the solutions with a different cocaine concentration were added to **S3**. After 20 min, the rhodamine B released from the pore voids of the support was measured by fluorescence spectroscopy. As seen in Fig. 4, the amount of rhodamine B delivered was proportional to the cocaine concentration in the solution. From this figure, a limit of detection (LOD) of ca. 5 × 10^−7^ M was determined. Both the LOD and analysis time for probe **S3** fell within the ranges of other methods reported for cocaine detection (see Table 2).

**Figure 5:**
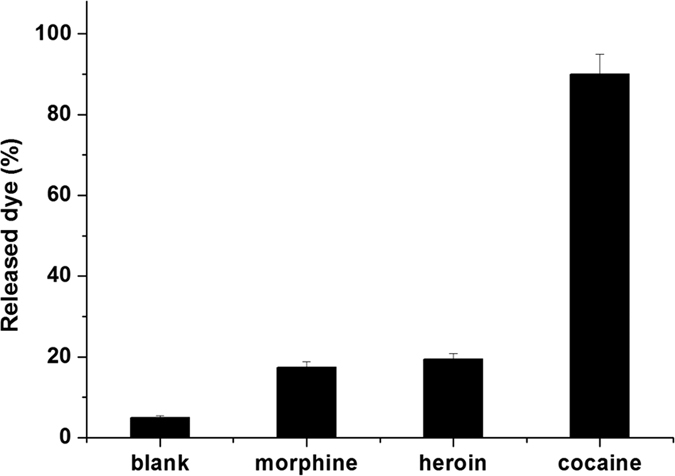
Release of rhodamine B from support **S3** in the presence of morphine, cocaine and heroin at 0.01 mM in Tris-HCl buffer.

The selectivity in the detection of cocaine of support **S3** was investigated by carrying out similar delivery experiments in the presence of other drugs (Fig. 5). As seen, poor cargo delivery from **S3** was observed with the 0.01 mM concentration of heroin and morphine compared with the response obtained for cocaine.

The rapid cocaine detection in oral fluids is crucial in *in situ* drug abuse controls^56^. Encouraged by our results, we studied the potential use of **S3** to detect cocaine in more competitive real samples. Thus the possibility of detecting cocaine in oral fluids, such as human saliva, was studied (vide infra). The typical cocaine concentration in saliva in real-life samples is highly variable because its concentration is related with the degree of abuse of this drug by consumers. Nevertheless, on the market there are several kits to detect cocaine drug consumption. The most widely used test employed by trained police is the Cozart® RapiScan (CRS) test, which is not specifically selective for cocaine, but for a family of opiates. This test has a cut-off point of 30 ng/mL (9.89 × 10^−8^ M)^57^,^58^ which is comparable with our LOD, and suggests that our method could be used for the selective detection of cocaine in saliva samples at typical concentration levels. In order to evaluate the possible use of our material for detection in realistic environments solids **S3** were submerged into different aliquots containing 100 μL of saliva spiked with cocaine (63 μM). Then, different known amounts of cocaine were added to each aliquot until final concentrations of 0.05, 0.1, 0.15 and 0.20 mM were reached. After 20 min, the emission of the rhodamine B released from solid **S3** in the different aliquots was measured and a calibration curve was obtained (see Figure S2 in the Supporting Information) that responds to the linear equation y = 4010212x + 227496. From the intercept of the curve with the x-axis a concentration of cocaine in the spiked sample of 56 μM was determined. Taking into account that the initial saliva sample had a cocaine concentration of 63 μM a 90% recovery was achieved.

Finally, we also tested the possible re-use of the NAA support. In this respect, an already used **S3** support was calcined to remove organic matter and was then functionalized following a similar procedure to that described above. Cargo release from recycled gated supports **S3-R** was very similar to **S3** (Figure S3). Moreover, calibration curve of solid **S3-R** was also very similar to that found for **S3** in terms of linear range and limit of detection (Figure S4). This recycled procedure via calcination was repeated 3 times with similar results (Figure S4). Both experiments demonstrated that the supports could be reused after cocaine detection.

In summary, we demonstrated that the combination of nanoporous anodic alumina supports and selected aptamers can be used to prepare gated probes to detect specific molecules. In particular, we loaded NAA with rhodamine B and capped the support using a specific oligonucleotide sequence capable of recognizing cocaine. The studies demonstrated that the functional support was able to retain the cargo in an aqueous buffered solution, yet a clear delivery of the entrapped dye was selectively observed in the presence of cocaine. The response of the gated support to different cocaine concentrations was evaluated and a LOD of 5 × 10^−7^ M was determined. Furthermore, the selectivity of the system was assessed, which demonstrated that heroin and morphine drugs were unable to induce remarkable dye delivery. In addition, the real applicability of capped support **S3** was confirmed by the detection of cocaine in a competitive matrix, such as saliva. As a final point, we also demonstrated that it was possible re-use the NAA support. Our procedure, based on measuring an easy-to-detect fluorescent molecule that is delivered when cocaine is present, avoids analyte pre-treatment steps, such as extraction or derivatization. Therefore, it is appropriate for rapid analyses by non-specialized personnel. We demonstrate that NAA is a suitable support to prepare optical gated probes with a synergic combination of the favorable features of selected gated sensing systems and NAA, which is easy to prepare and handle, and can be easily recovered.

## Methods

### General techniques

PXRD measurements were taken by a D8 Advance diffractometer using Cu Kα radiation (Philips, Amsterdam, The Netherlands). A Field Emission Scanning Electron Microscopy (FESEM) analysis was performed under a ZEISS Ultra 55 microscope. Fluorescence spectroscopy was carried out in a Felix 32 Analysis, version 1.2 (Build 56), PTI (Photon Technology International) instrument. Thermogravimetric analyses were carried out on a TGA/SDTA 851e balance (Mettler Toledo, Columbus, OH, USA), using an oxidising atmosphere (air, 80 mL min^−1^) with a heating program: gradient of 393–1,273 K at 10 °C min^−1^, followed by an isothermal heating step at 1,273 °C for 30 min.

### Chemicals

(3-isocyanatopropyl) triethoxysilane, rhodamine B, tris (hydroxymethyl) aminomethane (Tris), hydrochloric acid were purchased from Sigma-Aldrich Química (Madrid, Spain) and oligonucleotides **O1** (NH_2_-(CH_2_)_6_-5′-AAA AAA CCC CCC-3′), **O2** (TTT TGG GGG GGG GAG ACA AGG AAA ATC CTT CAA TGA AGT GGG TCT CCA GGG GGG TTTT-3′) and **O3** (TTT TGG GGG GAC CAC AAG ACA TGC ATC CCG GGG GGG TTTT) were acquired from Isogen-Lifesciences (Barcelona, Spain). All the products were used as received. All the drugs were provided by the Agencia Española del Medicamento y Productos Sanitarios (AEMPS; Spanish Agency of Medication and Health Products).

### Fabrication of the nanoporous anodic alumina (NAA) support

Porous alumina substrates were produced by electrochemical anodisation of high purity aluminium sheets (99.99% purity). The used electrolyte was sulphuric acid 0.3 M. Before anodisation, the aluminium sheets were electropolished in a mixture of ethanol and perchloric acid 4:1 (v:v) at 20 V for 4 min to reduce their surface roughness. Then sheets were cleaned with abundant water and ethanol, and air-dried to avoid any acid residue. The electropolished aluminium sheets were then anodised in the H_2_SO_4_ electrolyte by a two-step anodisation process. The first anodisation step was performed for 24 h at 10 V. The electrolyte temperature was 2 °C. This porous alumina layer was dissolved by wet chemical etching in a mixture of phosphoric acid 0.4 M and chromic acid 0.2 M at 70 °C for 3 h to obtain a pre-patterned aluminium surface. Subsequently, the second anodisation step was performed under the same anodisation conditions as the first step. The anodisation time for this second step, which determines layer thickness, was adjusted to produce a porous alumina layer with a thickness of 8 μm.

### Synthesis of support S1

For a typical synthesis, NAA was immersed in 8 mL of a mixture of rhodamine B (18.6 mg, 0.04 mmol) in CH_3_CN (30 mL). The suspension was stirred at room temperature for 24 h. Then excess (3-isocyanatopropyl) triethoxysilane (328 μl, 1.32 mmol) was added, and the final mixture was stirred at room temperature for 24 h. The resulting pink support (**S1**) was washed slightly with acetonitrile and dried at 37 °C for 2 h.

### Synthesis of support S2

Support **S1** was immersed in 700 μL of a solution of rhodamine B in CH_3_CN (1 mM). Then 100 μL of oligonucleotide **O1** (at 20 μM concentration) and 2 μL of triethylamine were added. Finally, the mixture was stirred 3 h at room temperature.

### Synthesis of support S3

Support **S2** was immersed in a solution that contained 780 μL of hybridisation buffer (20 mM Tris-HCl, 37.5 mM MgCl_2_, pH 7.5) and 41.05 μL of **O2** (100 μM). The mixture was stirred for 2 h at room temperature. The resulting material was thoroughly washed with hybridisation buffer (20 mM Tris-HCl, 37.5 mM MgCl_2_, pH 7.5) to eliminate the unbounded oligonucleotide.

### Synthesis of support S4

Support **S2** was immersed in a solution that contained 780 μL of hybridisation buffer (20 mM Tris-HCl, 37.5 mM MgCl_2_, pH 7.5) and 41.05 μL of **O3** (100 μM). The mixture was stirred for 2 h at room temperature. The resulting material was thoroughly washed with hybridisation buffer (20 mM Tris-HCl, 37.5 mM MgCl_2_, pH 7.5) to eliminate the unbounded oligonucleotide.

### Release experiments of supports S3, S3-R

To investigate the gating properties of **S3**, two fractions of this material were immersed in 2.5 mL of hybridisation buffer (20 mM Tris-HCl, 37.5 MgCl_2_, pH 7.5). Then 2.5 mL of a cocaine aqueous solution (1 mM) were added to one of the supports, whereas 2.5 mL of water were added to the other support. Both experiments were maintained at 25 °C and fractions were taken at certain times. Cargo release to the solution was measured by the rhodamine B fluorescence at 585 nm (λ_ex_ = 555 nm).

### Calcination of S3

Calcination of support **S3** was performed in a MC/1300 oven (Gallur) using a heating gradient from 25 °C to 550 °C (3° per min), followed by an isothermal period at 550 °C for 5 h.

### Detection of cocaine in saliva samples

For these experiments, 100 μL of a cocaine problem solution were added to 2,000 μL of free saliva until a final concentration of 0.063 mM. Then the drug was determined by the method of standard addition. rhodamine B fluorescence was monitored after 20 minutes.

## Additional Information

**How to cite this article**: Ribes, À. *et al*. Molecular gated nanoporous anodic alumina for the detection of cocaine. *Sci. Rep.* **6**, 38649; doi: 10.1038/srep38649 (2016).

**Publisher's note:** Springer Nature remains neutral with regard to jurisdictional claims in published maps and institutional affiliations.

## Supplementary Material

Supplementary Information

## Figures and Tables

**Figure 1:**
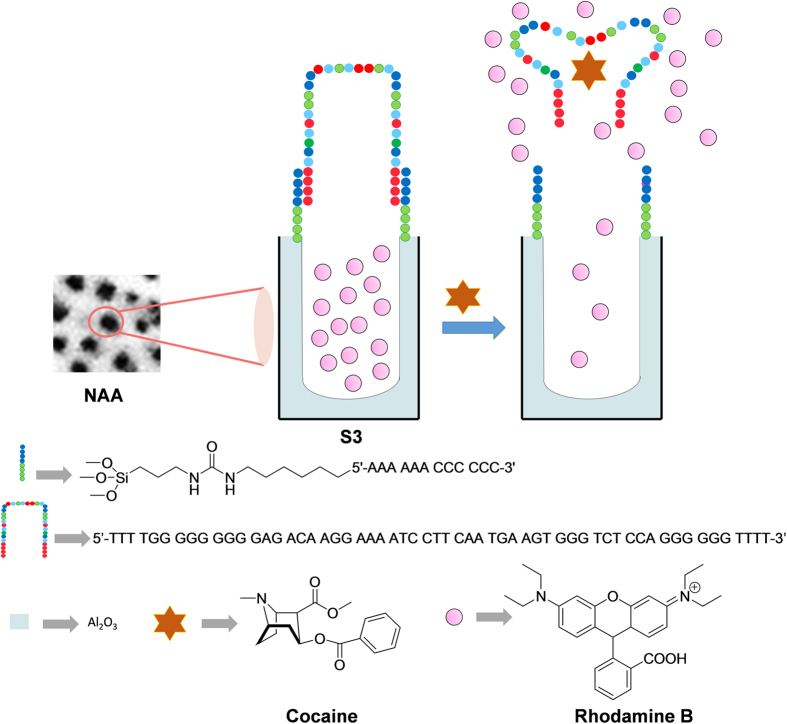
Scheme of the gated NAA support **S3** capped with the selected aptamer. Delivery of the entrapped dye (rhodamine B) is selectively accomplished in the presence of cocaine.

**Table 1:** Contents (in mmol/gSiO_2_) of (3-isocyanatopropyl), rhodamine B, **O1** and **O2** in the different hybrid supports.

	3-(isocyanatopropyl)	Rhodamine B	O1	O2	O3
S1	0.01	0.67			
S2	0.01	0.25	0.10		
S3	0.01	0.24	0.10	0.11	
S4	0.01	0.22	0.10		0.16

**Table 2:** Comparison of the LOD and analysis time of other methods reported to detect cocaine.

Sensory system	Detection	LOD (M)	Time (min)	Reference
Gas chromatography	Mass spectrometry	1.65 × 10^−11^	20	50
Cozart® RapiScan	Colorimetry	9.89 × 10^−8^	3	57, 58
Germanium strip waveguide on a silicon substrate integrated with a microfluidic chip	Infrared	1.65 × 10^−3^	10	59
Immunochromatographic paper-based strip coupled with an OLED	Fluorescence	1.64 10^−8^	Not reported	60
Microfluidic Electrochemical Aptamer-based Sensor	Voltammetry	10 × 10^−6^	20	51
Gold electrode functionalized with an aptamer	Electrochemiluminescence	3.7 × 10^−12^	120	52
Aptamer with gold nanoparticles	Colorimetry	100 × 10^−6^	Not reported	53
Isothermal circular strand-displacement amplification and graphene oxide absorption	Fluorescence	190 × 10^−9^	10	61
NAA loaded with rhodamine B and capped with cocaine aptamer	Fluorescence	5 × 10^−7^	20	This paper
