# Supplementary material for: Molecular gated nanoporous anodic alumina for the detection of cocaine
Source: Sci Rep. 2016 Dec 7;6:38649. doi: 10.1038/srep38649 (PMC5141502; doi:10.1038/srep38649)
Supplement: Supplementary Information [file srep38649-s1.pdf]

Supplementary Information

Molecular gated nanoporous anodic alumina for the detection of cocaine

Àngela Ribes,[1,2] Elisabet Xifré-Pérez,[3] Elena Aznar,[1,2] Félix Sancenón,[1,2] Teresa Pardo,[1,2] Lluís F. Marsal,[3,*] and Ramón Martínez-Máñez[1,2,*]

[1]Instituto Interuniversitario de Investigación de Reconocimiento Molecular y Desarrollo Tecnológico (IDM). Universitat Politècnica de València, Universitat de València, Departamento de Química, Universitat Politècnica de València, Camino de Vera s/n, 46022, Valencia, Spain
[2]CIBER de Bioingeniería, Biomateriales y Nanomedicina (CIBER-BBN)
[3]Departamento de Ingeniería Electrónica, Eléctrica y Automática, Universidad Rovira i Virgili, Avda. Països Catalans 26, 43007, Tarragona, Spain
[*]rmaez@qim.upv.es
[*]lluis.marsal@urv.cat

Powder X-Ray diffration

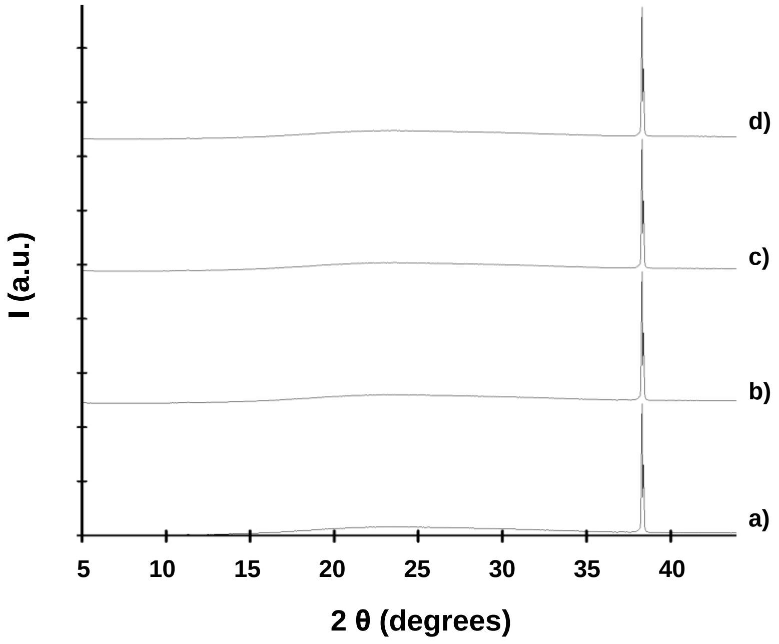

Figure S1. Powder X-ray diffraction pattern for a) as synthesized NAA support, b) **S1**, c) **S2** and d) gated **S3.**

Detection of cocaine in saliva samples

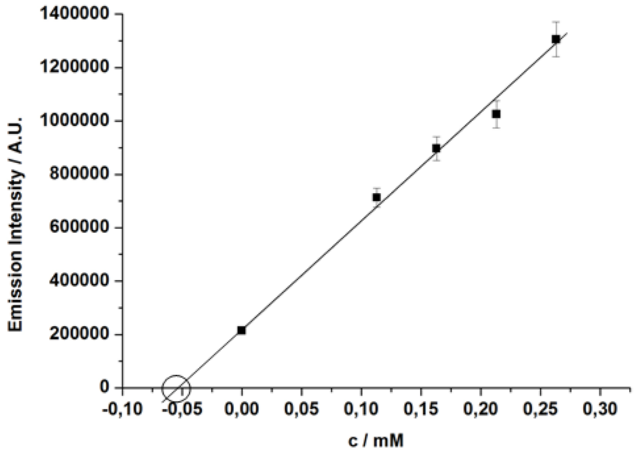

Figure S2. Standard addition method using **S3** for the detection of cocaine in a saliva sample spiked with cocaine (63 μM). From the intercept of the curve with the x-axis a concentration of cocaine of 56 μM was determined.

Release experiments of S3-R after calcination

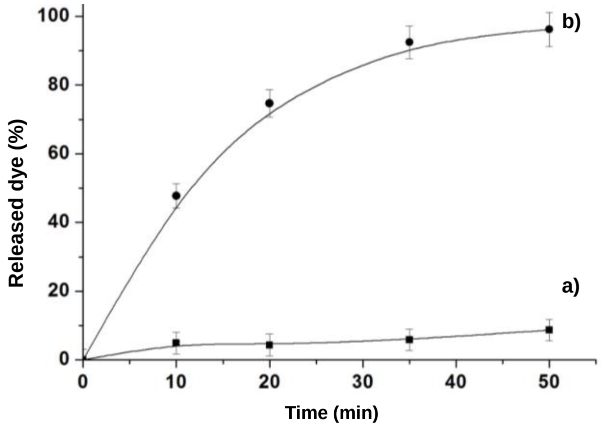

Figure S3. Release of rhodamine B from support **S3-R** in the absence (a) and in the presence (b) of cocaine (1 mM).

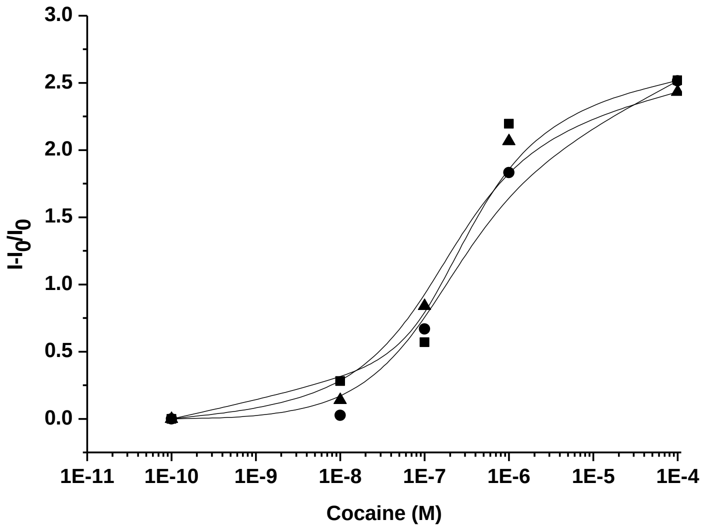

Figure S4. Release profiles of solid **S3-R** in the presence of increasing quantities of cocaine for three successive recycling calcination processes.
